# Assessment of Chicken Fecal Contamination Using Microbial Source Tracking (MST) and Environmental DNA (eDNA) Profiling in Silway River, Philippines

**DOI:** 10.3390/jox14040104

**Published:** 2024-12-12

**Authors:** Lonny Mar Opog, Joan Cecilia Casila, Rubenito Lampayan, Marisa Sobremisana, Abriel Bulasag, Katsuhide Yokoyama, Soufiane Haddout

**Affiliations:** 1 Agricultural and Biosystems Engineering Department, College of Agriculture, Mindanao State University, General Santos City 9500, Philippines; lonnymar.opog@msugensan.edu.ph; 2 Land and Water Resources Engineering Division, Institute of Agricultural and Biosystems Engineering, College of Engineering and Agro-Industrial Technology, University of the Philippines Los Baños, Laguna 4031, Philippines; rmlampayan@up.edu.ph; 3 School of Environmental Science and Management, University of the Philippines Los Baños, Laguna 4031, Philippines; 4 University of the Philippines Rural High School, College of Arts and Sciences, University of the Philippines Los Baños, Laguna 4033, Philippines; asbulasag@up.edu.ph; 5 Department of Civil and Environmental Engineering, Tokyo Metropolitan University, Hachioji 192-0397, Tokyo, Japan; k-yoko@tmu.ac.jp; 6 Department of Physics, Faculty of Science, Ibn Tofail University, Kenitra 14000, Morocco; soufian.haddout@gmail.com

**Keywords:** chicken fecal contamination, microbial source tracking, eDNA profiling, fecal contamination, flow velocity, water quality, Silway River

## Abstract

The Silway River has historically failed to meet safe fecal coliform levels due to improper waste disposal. The river mouth is located in General Santos City, the tuna capital of the Philippines and a leading producer of hogs, cattle, and poultry. The buildup of contaminants due to direct discharge of waste from chicken farms and existing water quality conditions has led to higher fecal matter in the Silway River. While there were technical reports in the early 2000s about poultry farming, this is the first study where fecal coliform from poultry farming was detected in the Silway River using highly sensitive protocols like qPCR. This study characterized the effect of flow velocity and physicochemical water quality parameters on chicken fecal contamination. Gene markers such as Ckmito and ND5-CD were used to detect and quantify poultry manure contamination through microbial source tracking (MST) and environmental DNA (eDNA) profiling. The results of this study showed the presence of chicken fecal bacteria in all stations along the Silway River. The results revealed that normal levels of water quality parameters such as temperature, pH, and high TSS concentrations create favorable conditions for chicken fecal coliforms to thrive. Multiple regression analysis showed that flow velocity and DO significantly affect chicken fecal contamination. A lower cycle threshold (Ct) value indicated higher concentration of the marker ND5-CD, which means higher fecal contamination. It was found that there was an inverse relationship between the Ct value and both velocity (R^2^ = 0.55, *p* = 0.01) and DO (R^2^ = 0.98, *p* = 0.2), suggesting that low flow velocity and low DO can lead to higher fecal contamination. Findings of fecal contamination could negatively impact water resources, the health of nearby residents, and surrounding farms and industries, as well as the health and growth of fish.

## 1. Introduction

Domestic chickens are common around the world, especially in the Philippines. They are one of the most essential sub-sectors of the Philippines’ agricultural industry. Chickens are typically the primary source of income in rural nations [1]. Consumers have a much greater preference for chicken meat, which has resulted in its being one of the most consumed meats due to its affordability. Among all the animals raised primarily for human consumption, one of the most consumed were chickens, which significantly contribute to the country’s livestock production volume.

**Figure 1:**
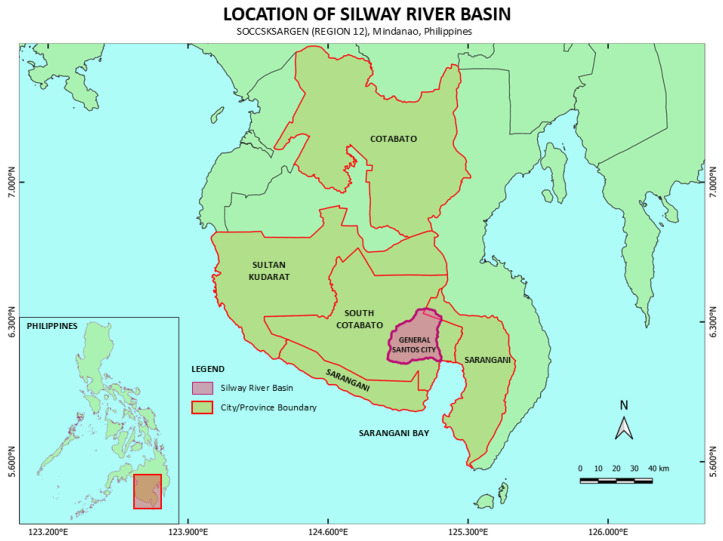
Location of Silway River basin in Southern Mindanao, Philippines.

According to the Philippine Statistics Authority’s situation report for the period October to December 2022, total chicken production was estimated at 494.12 thousand metric tons (live weight). Concurrently, the national chicken inventory was reported to be approximately 188.48 million fowls. Among the 18 regions in the Philippines, Region 12, also known as SOCCSKSARGEN, is derived from the provinces and cities that make up the region—South Cotabato, Cotabato, Sultan Kudarat, Sarangani, and General Santos City (Figure 1). The region ranks seventh among the major chicken-producing areas in the Philippines, contributing approximately 22,249 metric tons (live weight) to total chicken production. Additionally, it accounts for an estimated 10.37 million fowls in the national chicken inventory [2]. According to the Philippine Bureau of Animal Industry report, as of December 2022, there were a total of one hundred and eighty (180) registered poultry farms in South Cotabato.

The increasing demand for poultry products has raised waste management as a prominent issue within environmental considerations. One of the major sources of waste that contribute to environmental pollution is the chicken industry [3,4]. According to the local government, one of the issues experienced in this industry is the unregulated waste disposal methods and practices of poultry owners, which has resulted in various issues, particularly for the environment [3]. Chicken feces are released into the environment and receiving bodies of water as a result of commercial and backyard poultry raising, particularly during storms and floods [5].

Poultry fecal contamination poses sanitary risks by introducing pathogens like Escherichia coli, Salmonella spp., Campylobacter spp., and avian influenza viruses into water systems. Coliform bacteria, while useful indicators, do not directly reflect pathogen presence [6]. These microorganisms can cause gastrointestinal illnesses, systemic infections, and zoonotic outbreaks. Nutrient enrichment from poultry waste also promotes eutrophication, prolonging pathogen survival and fostering harmful algal blooms [7]. Addressing these risks is vital to safeguard water quality and public health.

According to research by the Department of Environment and Natural Resources, there are 180 out of 421 rivers in the country that are dirty and polluted and may soon be declared “biologically dead” [8], while 50 rivers are already considered “biologically dead”. Due to this situation, the local government agency identified several rivers in the country as Water Quality Management Areas in accordance with the Philippine Clean Water Act of 2004. This study aligns with the United Nations Sustainable Development Goal (SDG) 6, “Clean Water and sanitation”, which aims to ensure the availability and sustainable management of water and sanitation for all. With respect to SDG 14, “Life Below Water”, this work provides a novel tool for detecting and managing water pollution, leading to improved water quality especially for Sarangani Bay. Sarangani Bay is a significant source of income for the Sarangani province, and General Santos City, the tuna capital of the Philippines, drains to this water body. By employing an innovative approach to environmental monitoring and management, this work enhances water quality by reducing pollution, minimizing fecal contamination, and significantly increasing the availability of safe water reuse on a global scale.

Traditionally, fecal indicator bacteria (FIB) have been used to identify and count the presence of fecal water pollution, including fecal coliforms and enterococci [5], but due to a lack of host specificity related to genetic markers shared universally across a diverse range of hosts, FIB analysis is unable to provide information on possible sources of contamination [9]. In this study, a microbial source tracking (MST) method was used by focusing on host-specific mitochondrial DNA (mtDNA) of chicken to determine the fecal contamination in the Silway River. MST refers to a set of methodologies used to identify and, in some cases, quantify the primary sources of fecal contamination in various water bodies, including drinking water, groundwater, and recreational waters. These methods are classified into library-dependent and library-independent approaches, each designed to trace contamination to its origin [10].

Environmental DNA (eDNA) was used in the Philippines for other applications like metabarcoding and fish visual census [11]. In the Laguna Lake, Philippines, MST using rep-PCR markers was used for fecal contamination studies [12,13]. In other countries, microbial and chemical source tracking markers were used to monitor fecal contamination at drinking water intakes [14]. In China [5] and Virginia, USA [15], eDNA and MST were used for quantitative detection of domestic poultry feces and human and poultry fecal contamination, respectively.

In 2010, the Sustainable Sanitation for East Asia (SuSEA) Philippines component developed the Water Pollution Prevention and Control Program (WPPCP) with the intended result of lowering the biochemical oxygen demand (BOD) loading of the Silway River in South Cotabato by 10% at the end of the year [16]. The program was developed with the desire to support the designation of the Silway River’s WQMA. The fact that the Silway River was declared one of the Water Quality Management Areas (WQMAs) implies that the river’s current state demands extensive protection and management by the local government and stakeholders. On 28 November 2022, the Green City Action Plan (GCAP) for General Santos City was published. It established a new target of 30% reduction on BOD load and fecal coliform in Sarangani Bay by 2040 (from the 2018 level). This 20% increase in the reduction goal indicates that the water quality deteriorated over the past years [17].

The novelty of this study lies in the fact that it is the first local study in the Philippines about the application of host-specific detection and quantification of chicken fecal contamination in a river system. This study could also be used in determining chicken fecal contamination in soil samples. This study would open new research, water quality monitoring technique, and potential local government projects to address chicken fecal contamination not only in rivers but also in various aquatic and terrestrial environments, especially in regions with high total chicken meat production in the country. Lastly, the results of this study could help in the assessment of the water quality of Silway River stations that are not covered by the Silway River Water Quality Management Area.

This study aims to examine the level of chicken fecal contamination in the Silway River, Philippines, on a spatial scale. Several critical water quality parameters that are crucial to monitoring and environmental management were also assessed. Specifically, this study characterized flow velocity and physicochemical water quality parameters; utilized two gene markers, Ckmito and ND5-CD, to detect and quantify poultry manure contamination; evaluated chicken fecal contamination using MST and eDNA profiling; and assessed the relationship between chicken fecal contamination and physicochemical water quality parameters. The methodology employed in this study can be utilized for rivers and water bodies worldwide, providing a valuable tool for environmental monitoring and management across various contexts and regions.

## 2. Materials and Methods

### 2.1. Site Description

One of the rivers under protection in the Philippines is the Silway River in Mindanao. The Silway River estuary flows through a densely populated residential area with commercial establishments [18]. The Silway River is located in the southern part of South Cotabato province and the western part of Sarangani province (Figure 1). The Silway River WQMA comprises the municipalities of Polomolok, Tupi, and T’boli and General Santos City. Subsequently, these areas’ wastewater discharges drain into the Silway River.

The river basin falls within fifty-nine barangay jurisdictions, distributed in the four abovementioned cities or municipalities, and drains into Sarangani Bay [19]. The water quality of the Silway river directly impacts the bay [17], which is a protected seascape and a renowned spawning habitat for tuna fingerlings. General Santos City is a highly urbanized city in South Cotabato. The Silway River mouth is located in this city, which is known as the tuna capital of the Philippines with a GDP that is highly dependent on fish catch. Agriculture and fisheries are the main drivers of General Santos City’s economy.

### 2.2. Water Sampling Materials and Data Collection Equipment

Different materials and equipment were used in this study. An electromagnetic current meter (INFINITY-EM, JFE Advantech, Nishinomiya, Japan) with a data logger and built-in compass was used to measure the flow velocity. A YSI ProDSS multiparameter was used to identify the actual coordinates of the sampling stations and measure DO, turbidity, and temperature. A leveling rod was used to determine the depth of the river. A digital pH meter was used to measure the pH.

### 2.3. Preliminary Fieldwork Preparation

**Figure 2:**
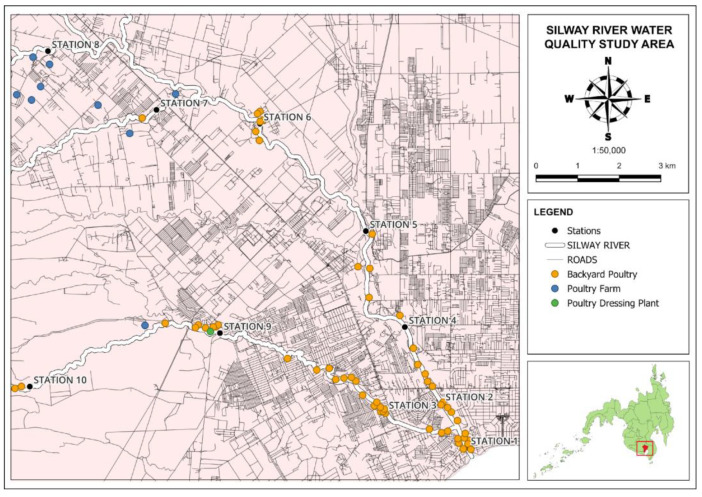
Sources of chicken fecal contamination and sampling locations along the Silway River tributaries in South Cotabato and General Santos City. The red square shows the location of Silway River basin.

A preliminary reconnaissance survey was conducted to identify the location of the sampling stations and nearby poultry farms and backyard poultries in Silway River. The initial coordinates of the stations were identified using Google Earth Pro. The selection of water sampling stations was determined based on the proximity to poultry farms and local communities engaged in backyard poultry raising and family poultry. Additionally, some stations were selected in alignment with the Silway River Water Quality Management Area (WQMA) stations. Figure 2 shows the map of the sources of chicken fecal contamination.

### 2.4. Methods for Water Sampling, Data Gathering, and Laboratory and Data Analysis

**Figure 3:**
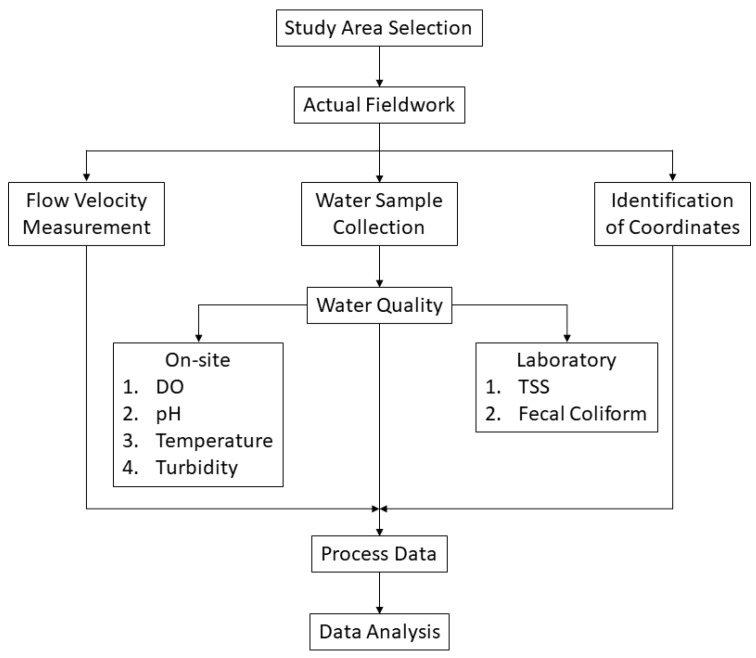
Procedural flow chart.

Figure 3 shows a flow chart with a brief procedural illustration of the study. Flow velocity, actual stations coordinates, water quality parameters, and water samples for laboratory tests were gathered simultaneously.

#### 2.4.1. Study Area Selection

**Figure 4:**
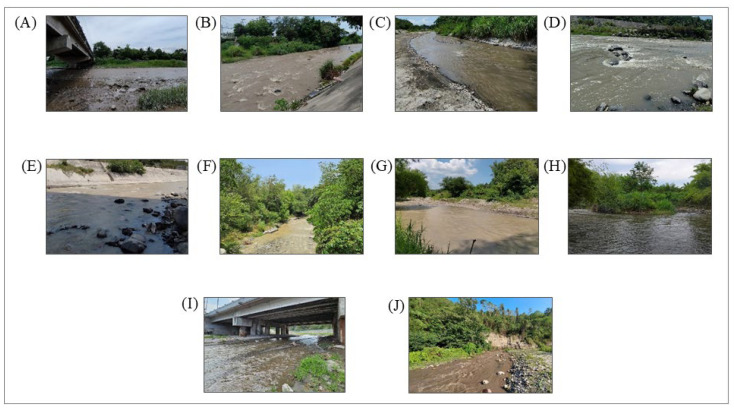
Images of the sampling locations: (**A**) Station 1, (**B**) Station 2, (**C**) Station 3, (**D**) Station 4, (**E**) Station 5, (**F**) Station 6, (**G**) Station 7, (**H**) Station 8, (**I**) Station 9, (**J**) Station 10.

The study area is located in the province of South Cotabato, a region in Southern Mindanao. The location of each sampling station was determined using an electromagnetic current meter with a data logger and built-in compass. The catchment area of the Silway River has a total of 56,280 hectares composed of different municipalities including Polomolok, with most of the portions and parts of T’boli, Tupi, and General Santos City. It is drained by several streams and tributaries that make up the 28 km Silway River [16]. Figure 4 shows the different sampling stations of the Silway River.

#### 2.4.2. Water Sample Collection and Data Gathering

The study was conducted on 14 April 2023 from 8:00 a.m. to 4:00 p.m. when the weather was sunny and clear. A total of 10 water samples were collected for this study from the sampling stations of Silway River, province of South Cotabato, including the different barangays of General Santos City namely, Labangal, Sinawal, City Heights, and San Isidro, and barangays of Polomolok including Silway 7 and Silway 8. Table 1 shows a list of the sampling stations and their descriptions.

All equipment and materials were rinsed with distilled water. Each sampling bottle was labeled with station number from one (1) to ten (10). Flow velocity was measured using an electromagnetic current meter (INFINITY-EM, JFE Advantech), while the pH was measured using a digital pH meter immediately after the samples were collected in each station. The water quality parameters such as dissolved oxygen (DO), temperature, and turbidity were measured using a multiparameter water quality meter (YSI ProDSS, Xylem Analytics, OH, USA) with an optical luminescence sensor for dissolved oxygen, thermistor sensor for temperature, and optical 90-degree scatter sensor for turbidity. Water quality parameter measurements were performed at a depth of 0.1 m in each station. The width of the river varies in every station, so it was divided into three sections to measure the flow velocity and depth, and these sections were averaged later for the profile distribution of velocity and depth.

#### 2.4.3. Laboratory Analysis

Water samples for laboratory tests were collected from the middle section of the river. A total of 1500 mL of the water sample was collected in each station, 500 mL for total suspended solid (TSS) tests, and 1000 mL for PCR and qPCR tests for chicken fecal coliform. After collection, water samples were properly sealed to prevent contamination and stored immediately in the insulated ice container to keep the trace elements. The gathered water samples were transported to the laboratories within 24 to 48 h from collection. The water samples for TSS were sent to the Mindanao State University—GSC Water Laboratory—while the water samples for fecal coliform analysis were sent to the Philippine Genome Center Mindanao Omics Laboratory.

The TSS analysis followed the EPA (1983) Method 160.2 (Gravimetric, Dried at 103–105 °C). Total suspended solid measurement for each station was obtained by filtration and gravimetric methods using a standard glass fiber filter paper, oven dryer, analytical weighing scale, and vacuum filtration. Initially, the filter paper was weighed to identify the initial weight. Then, each water sample was poured into the vacuum filtration set up. After allowing the water to be drained, the residue left on the filter paper was oven dried at a constant temperature of 105 °C for 48 h. Lastly, the filter paper was weighed to obtain the final weight, and the value for total suspended solids was determined using the following formula:

(1) $$TSS\;\left(\frac{mg}{L}\right)=\frac{W_{f}-W_{i}}{V}\times 1000000$$

where W_i_ = initial weight (g), W_f_ = final weight (g), and V = volume (mL).

For fecal coliform analysis, 1000 mL of water samples were processed by the Philippine Genome Center Mindanao Omics Laboratory Services. For the MST method, Ckmito and ND5-CD primers and probes were used for the PCR and qPCR tests. The nucleotide sequences were retrieved from the study conducted by [5,20]. Minor groove binder (MGB) probe and primers were synthesized by Noveaulab. The oligonucleotide sequences used are shown in Table 2.

First, environmental DNA (eDNA) was extracted from the water samples using the Qiagen PowerSoil Pro Kit (Qiagen, Hilden, Germany), adhering to the manufacturer’s instructions. Then, the concentration of the extracted eDNA in each station was measured using the spectrophotometer. After determining the concentration of the eDNA in every station, Agarose Gel Electrophoresis was performed with 1% Agarose Gel concentration and electrophoresed for 60 min at 100 V. For the detection of chicken fecal contamination, the samples were amplified using the Ckmito1 forward and reverse primer for single PCR with 20 μL of reaction volume and annealing temperature of 55 °C and were run in a conventional PCR machine. Gel concentration was 2% and electrophoresed for 90 min at 100 V. The same process was performed for the nested PCR test using CkmitoN1 forward and reverse primer. The amplified products were obtained using the primers Ckmito 1-G/D, and then the positive products were used as DNA templates when the primers CkmitoN1-G/D were applied. The markers Ckmito1 and CkmitoN1 were combined and collectively referred to as the Ckmito marker. To quantify chicken fecal coliforms, a TaqMan assay targeting the ND5 gene was conducted using a real-time qPCR system (Applied Biosystems, Foster City, CA, USA). Positive products from the nested PCR amplification were used as the DNA template to measure the cycle threshold (Ct) value.

**Figure 5:**
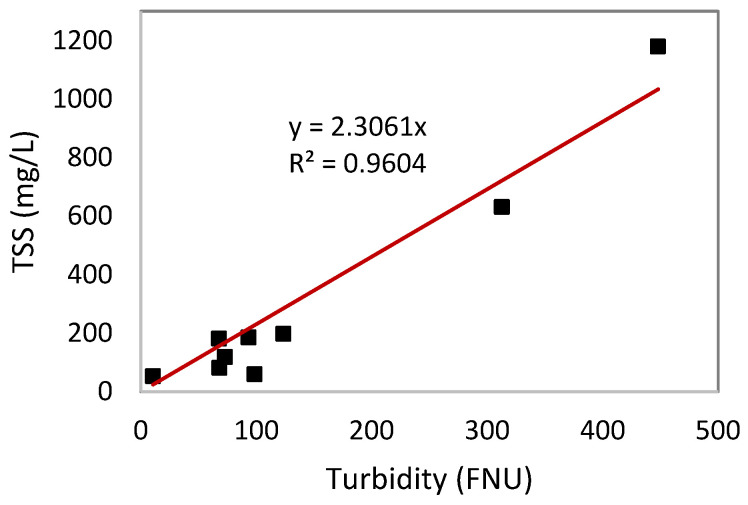
Regression analysis between turbidity (FNU) and analyzed total suspended solids (mg/L).

There were missing values for dissolved oxygen and turbidity that were observed from the report of the results and were later revealed as a result of instrumental error during the collection of samples in station 4. Although there were missing values, the researcher was able to estimate the turbidity measurement for station 4 using the conversion Equation (2) generated by performing linear regression using the linear relationship between analyzed suspended solids and turbidity from nine stations. Figure 5 shows the analysis for linear regression between turbidity (FNU) and analyzed TSS (mg/L).

(2) Turbidity=2.3061x

### 2.5. Data Mapping of Chicken Fecal Contamination and Physicochemical Water Quality Parameters Results

The coordinates of each sampling station were identified using the built-in compass in the electromagnetic current meter (INFINITY-EM, JFE Advantech, Nishinomiya, Japan). The data that were collected during this study and the results obtained from the water analysis were consolidated to generate a map for fecal contamination and water quality parameters using Quantum GIS (QGIS) software 3.32.3. The water quality guidelines for Class C freshwater primary parameters were also used to compare the results, whether they passed or failed the requirements.

## 3. Results and Discussion

### 3.1. Flow Velocity

Water quality (WQ) processes can be significantly influenced by flow velocity conditions. Specifically, higher flow velocities are associated with increased reaeration rates in a positive correlation [21]. The flow velocity of each station is shown in Table 3, including their depth and width. The values obtained for flow velocity ranged from 0.22 m/s at Station 9 to 1.27 m/s at Station 7. This was attributed to factors such as steep gradient in the area and narrow channel section in Station 7, which is 8.40 m. On the other hand, Station 9 shows the lowest flow velocity of 0.22 m/s among the recorded stations. This is due to its wider channel section of 12.1 m with a moderate gradient in the area. Additionally, its depth of 0.13 m stands as the lowest depth among all the stations.

The variations in flow velocity along the Silway River can be attributed to several factors, including the river’s topography, channel characteristics, and the volume of water being transported. The speed at which water moves through a river may influence several factors and parameters such as dissolved oxygen, suspended solids, temperature, nutrient transport, and pollutants dilution and removal that contribute to its overall quality [22].

### 3.2. Physicochemical Water Quality Parameters and Chicken Fecal Contamination

**Figure 6:**
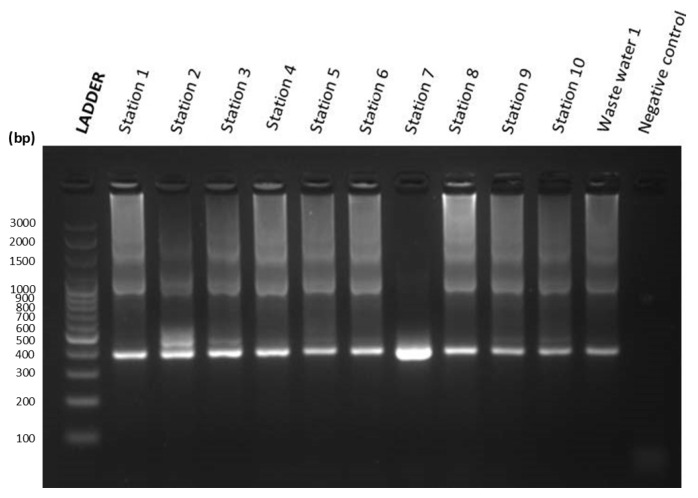
Gel image of first nested PCR of Silway River water samples.

**Figure 7:**
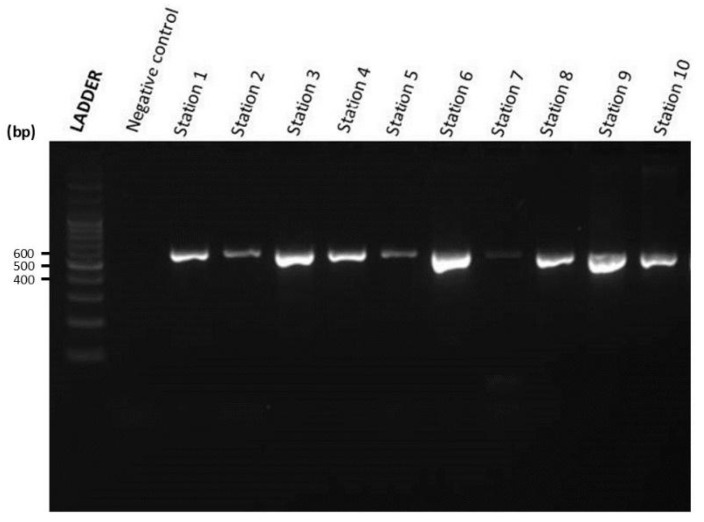
Gel image of second nested PCR of Silway River water samples.

The gel image results of the first and second nested PCR of Silway River water samples from Station 1 to Station 10 and a negative control are shown in Figure 6 and Figure 7. Figure 6 shows the agarose gel electrophoresis image result of the first nested PCR of water samples from Silway River stations. On the other hand, Figure 7 shows the agarose gel electrophoresis image result of the second nested PCR of water samples from Silway River stations.

The water quality parameters such as DO, TSS, temperature, pH, turbidity, and PCR and qPCR tests shown in Table 4 were based on the results from on-site fieldwork and the laboratory analysis conducted by the Philippine Genome Center Mindanao Omics Laboratory and Mindanao State University GSC Water Laboratory. The comparison between the results of water quality parameters analysis from ten sampling stations and the standards outlined in the DENR Administrative Order series of 2016-08 are also shown in Table 4.

#### 3.2.1. Dissolved Oxygen (DO)

**Figure 8:**
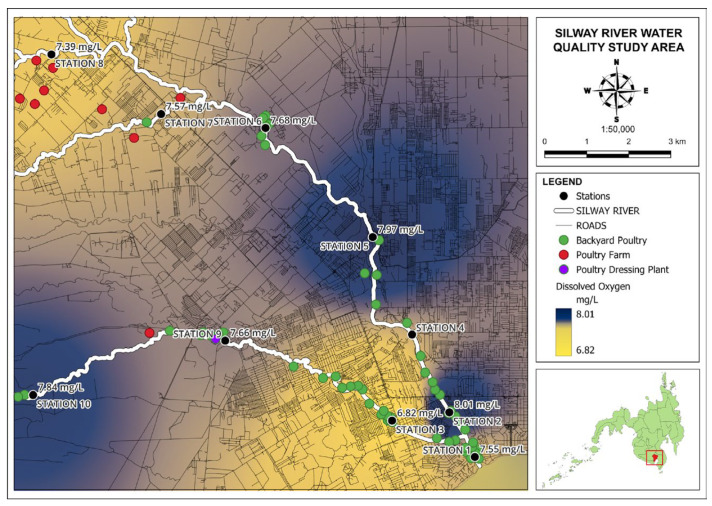
Map of dissolved oxygen results for Silway River stations.

The measurement of DO is crucial for assessing the metabolic pulses of rivers and is a fundamental indicator of water quality [22]. Figure 8 shows the map for the results of DO concentration along the Silway River.

In this study, Stations 2, 5, and 10 have relatively high DO levels with 8.01 mg/L, 7.97 mg/L, and 7.84 mg/L, respectively, while Station 3 has the lowest recorded DO level at 6.82 mg/L. All of the stations contain a DO concentration above the minimum level required, which means that most fish and other living organisms will be able to survive in the water. In the succeeding section, DO will be correlated with chicken fecal contamination.

#### 3.2.2. Total Suspended Solids (TSSs)

**Figure 9:**
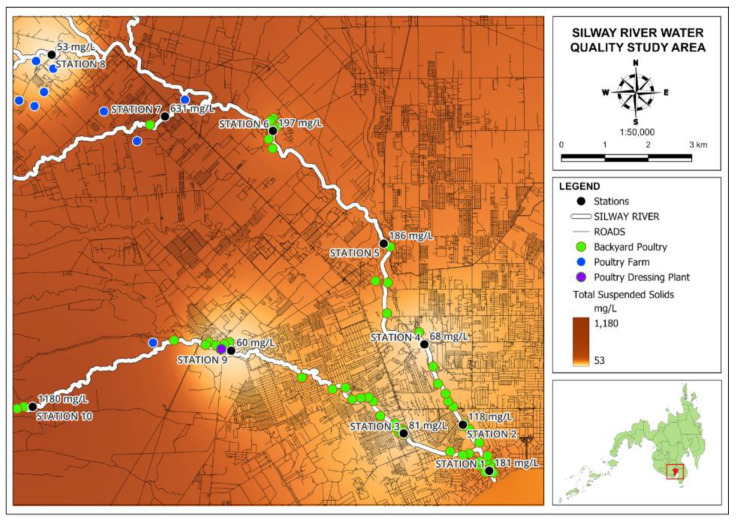
Map of total suspended solids in Silway River stations.

From the results shown in Table 4, the majority of the stations have TSS concentrations above the recommended limit of 80 mg/L for Class C freshwater. In Figure 9, Stations 7 and 10 have particularly high TSS levels, with 631 mg/L and 1180 mg/L, respectively. The high concentration of TSSs in the majority of the stations was due to their topography, where the area is characterized by hills and runoff. This is very common, like in Station 10, which is located at the bottom of a hill. On the other hand, only Stations 4, 8, and 9 pass the maximum threshold of 80 mg/L with 68 mg/L, 53 mg/L, and 60 mg/L, respectively. Besides the topographic conditions, flow velocity can also influence the transport and distribution of suspended solids in a river. Historically, in the Silway Water Quality Management Report 2020, 9 out of 25 monitoring stations failed the standard allowable TSS level. This poses a threat to environment safety and health hazards to nearby communities. The accumulation of sediment deposits at river channels not only harbors toxic pollutants but also diminishes the flood-carrying capacity [23].

#### 3.2.3. Temperature

The temperature of the water plays a crucial role in shaping the habitat availability for various aquatic species, thereby affecting the survival of some aquatic organisms. It has a significant impact on water quality and various environmental factors, as it governs the diversity of aquatic life, regulates the maximum concentration of dissolved oxygen, and influences the occurrence of chemical and biological reactions [8]. The readings for temperature in the Silway River reveal that the majority of the stations along the river fall within the acceptable temperature range. This indicates that the Silway River generally meets the water quality guidelines for primary parameters at these locations.

**Figure 10:**
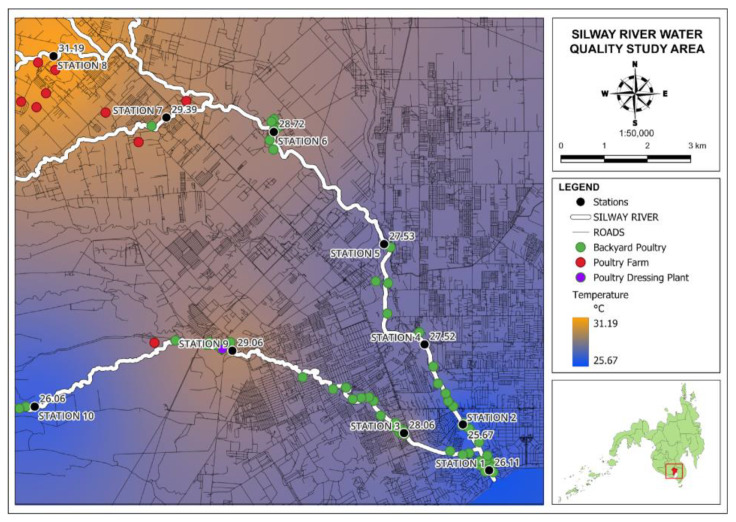
Map of temperature records in Silway River stations.

Stations 1 and 2 recorded temperatures of 26.11 °C and 25.67 °C, respectively; these values barely fall within the minimum limit. Station 8, by contrast, recorded a temperature of 31.19 °C, which is at the upper limit of the acceptable range. Figure 10 shows a map of the results of temperature.

#### 3.2.4. pH

**Figure 11:**
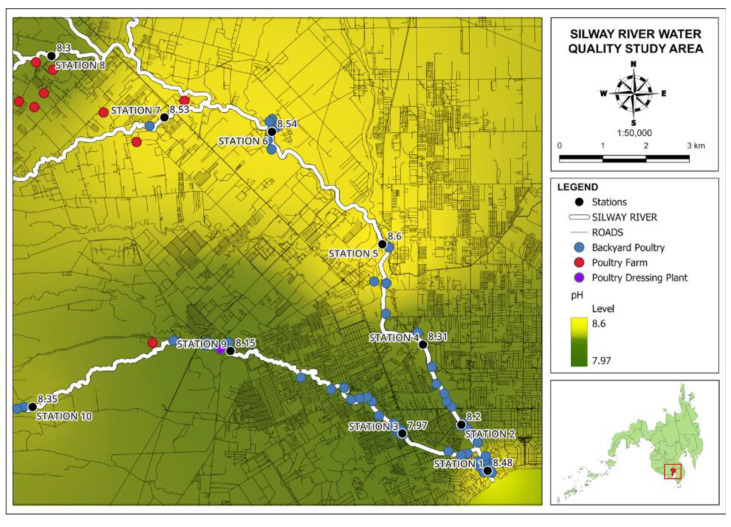
Map of pH levels in Silway River stations.

Low pH levels can lead to the release of toxic elements and compounds, including heavy metals, which can be absorbed by aquatic organisms. In this study, the pH levels recorded at the sampling stations in the given data were generally within the acceptable range, ranging from 7.97 in Station 3 to 8.60 in Station 5. These values indicate that the water in the Silway River is predominantly alkaline in nature. Fluctuations in pH can have detrimental effects on certain species in the river, while others may be more sensitive to such changes. Therefore, monitoring the pH of an aquatic system is essential for assessing the quality of water and pollution levels [8]. Figure 11 shows a map of the results of pH level.

#### 3.2.5. Turbidity

**Figure 12:**
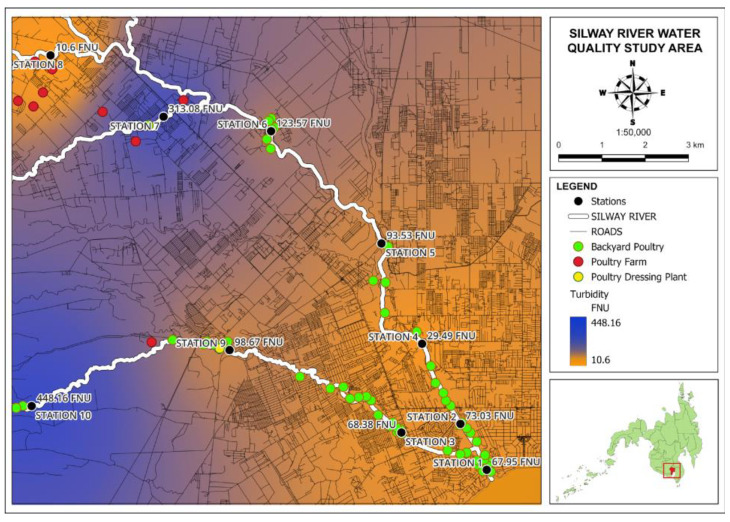
Map of turbidity values in Silway River stations.

As shown in Table 4, Station 10 records the highest turbidity value of 448.16 FNU, indicating an extremely high concentration of suspended particles and potentially very cloudy or muddy water. On the other hand, Stations 4 and 8 exhibit lower turbidity readings, with Station 8 having the lowest recorded value of turbidity at 10.60 FNU, suggesting clearer water with fewer suspended particles. Figure 12 shows a map of the turbidity of sampling stations in the Silway River.

Station 10 is an upstream hilly station with mainly pineapple plantations. Pineapple is a famous commodity in the area produced by contract farmers and in the area of a large international company. Research has shown that the rapid deterioration of soil biodiversity on pineapple plantations due to erosion, over-cultivation, and pesticide application eventually leave the land completely infertile [24,25]. Elevated turbidity levels can result in increased heat absorption by the water, leading to higher water temperatures. Additionally, the reduced light penetration caused by high turbidity can diminish photosynthetic activities of aquatic plants and organisms, consequently decreasing the level of dissolved oxygen in the water [26]. Station 7 had the second highest turbidity and had the second highest temperature during the time of data gathering. Station 3 had the lowest DO, and the turbidity level exceeded the minimum allowable standard.

#### 3.2.6. Chicken Fecal Contamination

The presence of fecal coliform in water has an adverse effect on the ecosystem and poses a substantial risk to human health [9]. In this study, two poultry-associated gene markers, mtDNA Ckmito and ND5-CD, were used as indicators for microbial source tracking of chicken fecal contamination. The mtDNA marker Ckmito was utilized for MST by PCR to detect chicken fecal contamination. The ND5 gene marker was used for the qPCR assay to quantify chicken fecal contamination. As shown in Table 4, all stations of the Silway River tested positive for chicken fecal contamination in the PCR tests, and all of the samples showed a reaction during the qPCR test. Table 5 further shows the number of poultry farms, backyard poultry, and dressing plants near the station.

**Figure 13:**
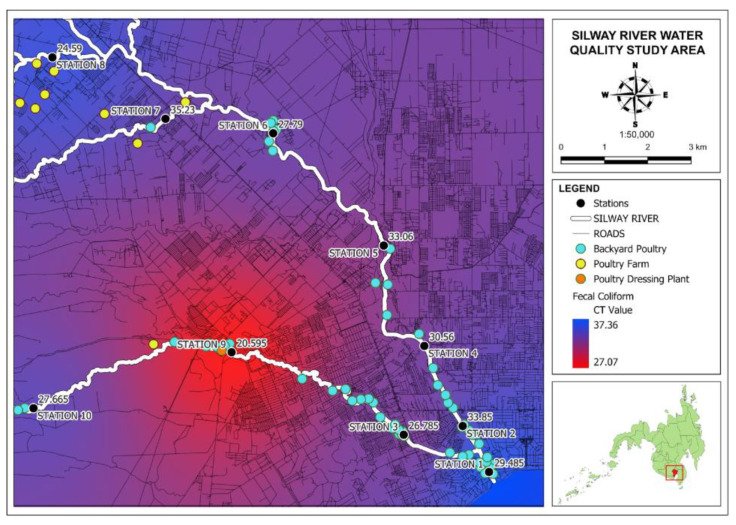
Map of chicken fecal contamination in Silway River stations.

The Ct (Cycle threshold) value is the number of amplification cycles (utilizing RT-PCR) necessary for the fluorescence of a PCR product to intersect a threshold level, exceeding the background signal [27]. For Ct values, the higher the number, the lower the target gene concentration, while lower Ct values indicate a higher concentration of the target gene. Figure 13 shows a map of the results of chicken fecal contamination in the Silway River.

Station 7 has the highest Ct value at 35.23, indicating a low concentration of the marker ND5, while Station 9 and 8 have the lowest Ct values of 20.56 and 24.25, respectively, indicating significantly high gene concentration. The results from PCR and qPCR tests were expected due to the nearby communities and facilities in the Silway River that engaged in poultry raising, especially in Station 9. Backyard poultry raising is very common in various neighborhoods, especially in the province.

The MST method was used in this study to detect and quantify chicken host-specific fecal bacteria using a combination of PCR and qPCR. Based on the results of this study, chicken feces were indeed present and are one of the various sources of fecal contamination that contributes to water pollution in the Silway River. MST is recommended for use especially in studies involving identification and quantification of a specific fecal bacteria not only in water bodies but also in soils. The advantage of MST is that this method has been proven by previous studies to accurately and precisely identify various host-specific sources of fecal contamination in environmental water samples [13]. The tests involved in this method are generally more sensitive and specific than traditional fecal coliform tests.

One of the disadvantages of the MST method is the cost of the tests, which are more expensive to set up, but this can be cost-effective in the long term due to their accuracy and specificity. Table 6 shows a comparison between the cost incurred for the tests in this study and the cost of a traditional fecal coliform detection test.

The cost for a traditional fecal coliform test is based on Mindanao State University—GSC Water Analytical Laboratory and Research Center, while the cost for the tests using the MST method is based on Philippine Genome Center Mindanao Omics Laboratory services. The primers and probe were synthesized by Noveaulab and cost about PHP 29,191.50 as a whole. These primers can analyze at least 700 samples, while probes can analyze at least 250 samples.

### 3.3. Relationship Among Flow Velocity, Physicochemical Water Quality Parameters, and Chicken Fecal Contamination

#### 3.3.1. Chicken Fecal Contamination and Physicochemical Water Quality Parameter Relationship

The rate of coliform bacteria decay tends to rise with higher water temperatures, increased pH levels, nutrient scarcity, and greater levels of predation and parasitism. On the other hand, the survival of coliform bacteria can be extended, or they can even thrive under specific environmental conditions, including appropriate pH, suitable temperature, abundant nutrients, and concentrated suspended particles [28,29]. In this study, the levels of temperature and pH fell within the recommended range, while the majority of the results of TSSs failed the requirements. As mentioned from previous studies, these levels of water quality parameters such as temperature and pH and high concentrations of TSSs can be considered favorable conditions for fecal coliforms to grow and survive. Due to this, coliforms coming from chicken feces can stay and survive longer in these environments and continue to multiply. Fecal contamination is significantly high and has always been a problem in the Silway River.

#### 3.3.2. Multiple Regression Analysis of Chicken Fecal Contamination, DO, and Flow Velocity

An inverse relationship was observed between chicken fecal contamination and both DO and flow velocity when examining the data of the Silway River (Table 7).

**Figure 14:**
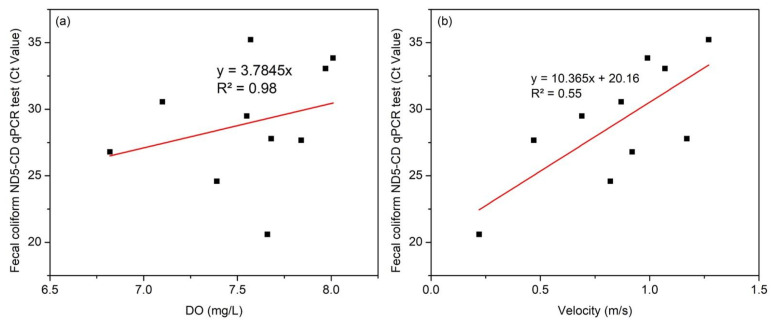
Relationship between fecal coliform Ct value and (**a**) DO (mg/L) and (**b**) velocity (m/s).

An R^2^ value of 0.98 indicates that higher DO has a higher Ct value, which means less chicken fecal contamination (Figure 14a). Low DO levels could indicate a higher presence of bacteria using it for respiration and an increased amount of decaying organic matter [30]. Meanwhile, an R^2^ value of 0.55 and a *p*-value of 0.01 indicate a significant effect of flow velocity on chicken fecal contamination (Figure 14b). With the availability of a current meter and DO meter, fecal coliform Ct value could be estimated using the equation Y = −6.920926245 + 3.571535586 × DO + 10.46724234 × Velocity. The equation developed is specific to the Silway River section studied on the given date, reflecting the conditions observed, and has not been validated or applied to other systems.

The concentration of chicken fecal contamination tends to increase as the flow velocity decreases. This trend was particularly evident in Station 9, which has the lowest Ct value of 20.595, indicating a significantly high concentration of fecal contamination, while flow velocity was lowest at 0.22 m/s. In addition, Station 7 has the highest flow velocity of 1.27 m/s while fecal contamination concentration was lowest with a Ct value of 35.230. This chicken fecal contamination and flow velocity relationship suggests that lower flow velocity may result in the accumulation of fecal contaminants, leading to higher concentrations. Lower flow velocity, particularly in stagnant water conditions, is more favorable for bacterial growth [31] and can promote the settling of fecal contaminants and particulate-bound pathogens in the riverbed. Historical data of the Silway Water Quality Management Report in the year 2020 showed that 25 out of 25 monitoring stations failed the standard allowable fecal coliform level of 200 MPN/100 mL. This means that the fecal coliform contamination problem has been an ongoing, persistent problem that must be addressed.

#### 3.3.3. Turbidity and TSS Relationship

There is a positive linear relationship between turbidity and TSSs. An R^2^ value of 0.961 and a *p*-value of <0.0001 indicates a highly significant effect in the relationship between turbidity and TSSs. As turbidity levels increase, TSS levels also increase. The equation y = 2.3061x was used as the turbidity–TSS conversion equation for all the turbidity data collected on site.

This trend is evident in all stations of the Silway River. Station 10 has the record of the highest values in both turbidity and total suspended solids concentration at 448.16 FNU and 1180 mg/L, respectively, and Station 7 is the second highest with a turbidity value of 313.08 FNU and total suspended solids concentration of 631 mg/L. By contrast, Station 8 has the lowest turbidity and total suspended solids concentration value of 10.60 FNU and 53 mg/L, respectively. This result was expected due to the presence of suspended solids such as organic matter and particles of sediment that cause light to scatter and be absorbed in the water, which can result in increased turbidity. In this study, the positive linear relationship between turbidity and TSSs implies that turbidity can be used as a proxy for estimating TSS levels.

## 4. Conclusions

Molecular analyses, such as qPCR using host-specific markers (ND5-CD, Ckmito) and MST methods, were used along with river water parameter analysis to investigate fecal contamination in the Silway River. The water samples collected from all stations tested positive for the Ckmito marker during the first and nested PCR tests, and all water samples exhibited a reaction crossing the threshold during the qPCR test. The results of this study suggest that nearby households engaged in backyard poultry raising and poultry farms are contributing to the chicken fecal contamination in the Silway River. In addition, the MST method combining Ckmito and ND5 markers proved effective in detecting and quantifying host-specific fecal bacteria in water bodies. However, the MST method is more expensive to set up, although it is considered cost-effective in the long term due to its accuracy and specificity.

Physicochemical parameters such as dissolved oxygen, temperature, and pH were all within the standard limit in all stations except for total suspended solids, where the majority of the sampling stations fell above the maximum limit. Although turbidity was not mentioned in the DENR standard water quality guidelines, the results in this study showed significantly high levels of turbidity. The DO levels in all stations were generally above the minimum required for aquatic life, which is >5 mg/L. The temperature fell within the acceptable range at all stations, but Station 8 was at the upper limit at 31.19 °C. pH levels were on the basic side, ranging from 7.97 to 8.60, indicating mainly alkaline water. TSS and turbidity levels were exceptionally high in Stations 7 and 10. These elevated TSS and turbidity levels are largely attributed to the topography of the study area, characterized by hills and runoff. This situation was evident in Station 10, which is located at the foot of a hill, suggesting cloudy or muddy water.

Relationships among flow velocity, water quality parameters, and chicken fecal contamination were also studied. Chicken fecal contamination and water quality parameters relationship suggests that normal levels of temperature and pH, along with high concentrations of TSSs, create a favorable condition for fecal coliforms to thrive. Chicken fecal contamination and flow velocity showed a significant inverse relationship, with an R^2^ value of 0.55 and *p*-value of 0.01, suggesting that lower flow velocity may result in the accumulation of fecal contamination, leading to higher concentrations and contributing to persistent fecal contamination in the Silway River. Similarly, DO had an inverse relationship with chicken fecal contamination since lower Ct values mean higher contamination. A strong coefficient of determination was found between DO and chicken fecal contamination Ct value, where R^2^ was 0.98 with a *p*-value of 0.2, suggesting that low DO means higher fecal contamination. Lastly, turbidity and TSS exhibited a strong positive linear relationship, with an R^2^ value of 0.961 and a *p*-value of <0.0001, such that higher turbidity levels were associated with elevated TSS levels. Turbidity can be used as a proxy for estimating TSS levels.

While direct relationships between fecal contamination and river water parameters were obtained, the molecular tools used were significant to pinpoint a specific fecal contributor, which is poultry. The results are essential for targeted remediation strategies. High-order riverine systems are influenced by multiple tributaries and diverse inputs, making it challenging to associate fecal contamination with individual upstream activities. MST and qPCR methods address this complexity by using source-specific markers, even in matrices with low fecal loads, where conventional approaches fail.

The results of this study were, however, derived from water samples collected during a single day under specific meteorological conditions. Environmental variables, such as prolonged drought or typhoon events, could substantially alter the outcomes. Drought conditions may reduce surface runoff, which could lead to undetectable levels of chicken markers. By contrast, typhoon-induced runoff could significantly elevate fecal contamination levels due to a high influx of water from more pollution sources. Therefore, these findings are limited by time and location and should be interpreted in the context of the environmental conditions during the sampling.

In conclusion, this study confirms the presence of chicken fecal contamination in the Silway River and emphasizes the significance of the application of the MST method for detecting and quantifying chicken-specific fecal bacteria in water bodies. With the growing accessibility of qPCR technologies, molecular analyses are pivotal for both addressing chronic water quality impairments and preventing contamination. This is particularly valuable, as communities face escalating challenges from both point and non-point pollution sources. Nearby households engaged in backyard poultry raising and poultry farms are one of the main sources of chicken fecal contamination in the Silway River. Lastly, flow velocity and water quality parameters play a significant role in the survival of certain fecal bacteria in environmental waters. The MST and eDNA approaches could be utilized in future research to assess the transport and persistence of pathogens in river systems, alongside hydrodynamic flow models, to offer a comprehensive understanding of contamination dynamics over time and space.

## Figures and Tables

**Table 1:** Silway River sampling stations.

Station	Latitude	Longitude	Station Description
1	6.1058	125.1640	Approx. 300 m from the Mouth of Silway River GSC
2	6.1154	125.1585	Approx 435 m from Silway Bridge along Digos-Makar Rd. at Purok Palen Brgy. Labangal GSC
3	6.1136	125.1462	Approx. 340 m from Sinawal Bridge along Makar—Gensan Rd. at Purok San Vicente Brgy. Labangal GSC
4	6.1321	125.1505	Foot of bridge along Yumang St. Purok Sta. Teresita Rd. City Heights GSC
5	6.1531	125.1420	Foot of Upper Silway Bridge along GSC Circumferential Rd. at Brgy. San Isidro GSC
6	6.1765	125.1190	Foot of Silway 7 Bridge along Silway 7—Klinan 6 Rd. at Silway 7 Polomolok
7	6.1795	125.0966	Approx. 250 m from Matin-ao Bridge of Purok Riverside Matin-ao Silway 8 Polomolok
8	6.1923	125.0730	Downstream portion of Silway River at Purok Upper Matin-ao Silway 8 Polomolok
9	6.1308	125.1103	Foot of Upper Sinawal Bridge along GSC Circumferential Rd. at Purok Cabuay Brgy. Sinawal GSC
10	6.11913	125.06902	Upstream portion of Silway River at Purok Kintay Brgy. Sinawal GSC

**Table 2:** Oligonucleotide primers and probes used for validation of MST qPCR and PCR assays.

Assay	Primer or Probe	Concentration in Final Reaction Mix	Oligonucleotide Sequence (5′-3′)	Product Size (bp)	Annealing T (°C)	Target Gene	Target Host
ND5-CD (TaqMan)	ND5-F	900 nM	ACCTCCCCCAACTAGC	172	60	Mitochondrial genes: NADH dehydrogenase subunit 5 (ND5)	Poultry (Chicken and duck)
	ND5-R	900 nM	TTGCCAATGGTTAGGCAGGAG				
	ND5-P	250 nM	(6-FAM ^1^) TCAACCCATGCCTTCTT (NFQ-MGB ^2^)				
Ckmito1 (single PCR)	Ckmito1-G	200 nM	ACCCTATTTGACTCCCTCAA	565	55	Mitochondrial 16S rRNA gene	Poultry (Chicken)
	Ckmito1-D	200 nM	ATGTCGACCAGGGGTTTATG				
CkmitoN1 (Nested PCR)	CkmitoN1-G	200 nM	CCCCCACACTAACAAGCAAT	381	55	Mitochondrial 16S rRNA gene	Poultry (Chicken)
	CkmitoN1-D	200 nM	GGTTGTAAGGTGGTCGTGAT				

^1^ 6-FAM: 6-Carboxy-Fluorescein. ^2^ NFQ-MGB: Non-Fluorescent Quencher-Minor Groove Binder.

**Table 3:** Velocity, depth, and width of the sampling stations in the Silway River.

Station Number	Velocity (m/s)	Depth (m)	Width (m)
1	0.69	0.55	23.5
2	0.99	0.48	22.9
3	0.92	0.27	6.4
4	0.87	0.54	28.3
5	1.07	0.30	16.5
6	1.17	0.52	24.6
7	1.27	0.35	8.4
8	0.82	0.62	16.9
9	0.22	0.13	12.1
10	0.47	0.35	8.0

**Table 4:** Physicochemical water quality parameters and PCR and qPCR test results for chicken fecal contamination.

Station Number	DO (mg/L)	TSS (mg/L)	Temperature (°C)	pH	Turbidity (FNU)	Fecal Coliform Ckmito PCR Test	Fecal Coliform ND5-CD qPCR Test (Ct Value)
DAO Standard	>5 mg/L	<80 mg/L	25–31	6.5–9.0	-	-	-
1	7.55	181	26.11	8.48	67.947	Positive	29.485
2	8.01	118	25.67	8.20	73.029	Positive	33.850
3	6.82	81	28.06	7.97	68.375	Positive	26.785
4	No data	68	27.52	8.31	29.487	Positive	30.560
5	7.97	186	27.53	8.60	93.532	Positive	33.060
6	7.68	197	28.72	8.54	123.575	Positive	27.790
7	7.57	631	29.39	8.53	313.084	Positive	35.230
8	7.39	53	31.19	8.30	10.599	Positive	24.590
9	7.66	60	29.06	8.15	98.666	Positive	20.595
10	7.84	1180	26.06	8.35	448.161	Positive	27.665

**Table 5:** Poultry farms, backyard poultry, and dressing plants near the stations in the Silway River.

Station Number	Number of Poultry Farm ^a^, Backyard Poultry ^b^, and Dressing Plants ^c^ Within a 1000 m Radius of the Station	Approx. Distance of Structures from the River (m)
1	12 ^b^	10–100
2	7 ^b^	10–80
3	7 ^b^	10–50
4	4 ^b^	10–40
5	3 ^b^	10–30
6	6 ^b^	10–60
7	3 ^a^, 1 ^b^	50–150
8	5 ^a^	200–300
9	1 ^a^, 6 ^b^, 1 ^c^	10–150
10	2 ^a^, 2 ^b^	10–100

^a^ Poultry Farm. ^b^ Backyard Poultry. ^c^ Dressing Plants.

**Table 6:** Cost comparison between MST and a traditional fecal coliform test.

Method	Unit	Price per Unit/Test
Traditional Test		
Detection of Fecal Coliform	samples	PHP 350.00
MST (detection only)		
eDNA Extraction (Water)	samples	PHP 976.60
Gel Electrophoresis	runs	PHP 158.92
PCR Amplification	amplicon	PHP 244.25
PCR Amplification Optimization	amplicon	PHP 244.25
Total		PHP 1624.12
MST (detection and quantification)		
eDNA Extraction (Water)	samples	PHP 976.60
Gel Electrophoresis	runs	PHP 158.92
PCR Amplification	amplicon	PHP 244.25
PCR Amplification Optimization	amplicon	PHP 244.25
qPCR	NC + PC	PHP 112.35
qPCR Optimization	gDNA + NC + PC + NEC	PHP 112.35
Total		PHP 3868.82

**Table 7:** Multiple linear regression analysis results.

Regression Statistics					
Multiple R		0.799465765			
R Square		0.63914551			
Adjusted R Square		0.536044227			
Standard Error		3.04281321			
Observations		10			
ANOVA					
	df	SS	MS	F	Significance F
Regression	2	114.7932244	57.3966122	6.199200363	0.028226849
Residual	7	64.8109856	9.258712228		
Total	9	179.60421			
	Coefficients		Standard Error	t Stat	*p*-value
Intercept	−6.920926245		20.75159251	−0.333513018	0.748514646
DO (mg/L)	3.571535586		2.709846892	1.317984273	0.229000056
Velocity	10.46724234		3.173606926	3.298216378	0.013152948

## Data Availability

The original contributions presented in the study are included in the article, further inquiries can be directed to the corresponding author.
